# Supplementary material for: Dietary Supplementation of Ancientino Ameliorates Dextran Sodium Sulfate-Induced Colitis by Improving Intestinal Barrier Function and Reducing Inflammation and Oxidative Stress
Source: Nutrients. 2023 Jun 19;15(12):2798. doi: 10.3390/nu15122798 (PMC10304453; doi:10.3390/nu15122798)
Supplement: Supplementary file 1 [file nutrients-15-02798-s001.zip › nutrients-2432756-supplementary.pdf]

**Supplemental Figure S1:**

1.1. Cell viability

Relative cell viability was measured using the cell counting kit 8 (CCK-8) assay. Caco-2 cells were dispensed in 96-well plates at $7\times10^3$ cells per well. After cell adherence, cells were administered with different dose of Ancientino (0, 12.5, 25, 50, 100 μg/mL) for 24 h. Then CCK-8 solution was added to each well and incubated at 37℃ for 4 h. The absorbance was measured at 450 nm to calculate the relative cell viability.

To investigate the anti-colitis activity of Ancientino *in vitro*, Caco-2 cells were selected for research. The cells were treated for 24 h with different dose of Ancientino (0, 12.5, 25, 50, 100 μg/mL) and the relative viability of the cells was measured. As shown in Fig S1, Ancientino-treated groups exhibited no significant difference on the relative cell viability compared to the control group. The results demonstrated that Ancientino has no apparent cytotoxicity to the Caco-2 cells. Based on these preliminary data 25, 50,100 μg/mL were selected in the succedent experiments.

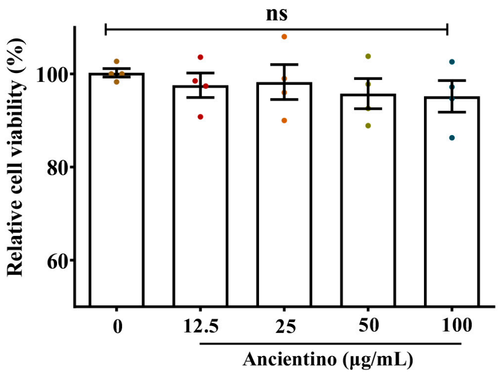


Figure S1. Cell viability. Cell viability is expressed as a percentage relative to control (Ancientino, 0 μg/mL). Every dot in the figure represents an independent experiment, and different colors represent different groups. Data are presented as the mean ± SEM, with data obtained from 4 independent experiments. ns indicates $p > 0.05$.
